# The relationship between tooth decay with stress and BMI among elementary students in Iran

**DOI:** 10.3389/fpubh.2022.920004

**Published:** 2022-08-30

**Authors:** Fatemeh Hosseinpour, Rahman Panahi, Baharan Ranjbar Omidi, Erfan Khorasani, Leila Dehghankar

**Affiliations:** ^1^Student Research Committee, School of Nursing and Midwifery, Qazvin University of Medical Sciences, Qazvin, Iran; ^2^Department of Health Education and Promotion, School of Medical Sciences, Tarbiat Modares University, Tehran, Iran; ^3^Department of Restorative Dentistry, School of Dentistry, Qazvin University of Medical Sciences, Qazvin, Iran; ^4^Student Research Committee, Qazvin University of Medical Sciences, Qazvin, Iran; ^5^Social Determinants of Health Research Center, Research Institute for Prevention of Non-communicable Diseases, Qazvin University of Medical Sciences, Qazvin, Iran

**Keywords:** tooth, health, student, stress - compressive, decay

## Abstract

**Background:**

Tooth decay is one of the most common chronic diseases among children worldwide. Stress and body mass index are also amongst the arguable risk factors which will affect people considerably. They include biological (hormones and blood sugar), socio-economic, and environmental factors and also lifestyle. In the present study, the relationship between tooth decay with stress and BMI in children was therefore investigated.

**Materials and methods:**

This was a cross-sectional study of a descriptive-analytical type. A total of 350 students who were referred to the clinic of the faculty of dentistry at Qazvin University of Medical Sciences during 2021–2022 were selected through convenience sampling method to participate in the study. First, the students underwent a dental examination after being measured on their height and weight. Then, two questionnaires; namely, demographic information and Children's Stress Symptom Scale (CSSS) of Scherer and Ryan-Wenger were completed by the children's parents through self-reporting. The collected data were analyzed using SPSS software version 23 and then descriptive statistics and logistic regression were applied.

**Results:**

The mean BMI of the participating students was in the normal range. The prevalence of tooth decay among participants was 76.9% (269 people). The mean and standard deviation of stress scores was 6.85 ± 4.01 out of 26, which was at a low level. Also, the mean and standard deviation of the BMI were 22.78 ± 5.28, which was within the normal range. The results of the logistic regression showed that the variables of “father's level of education,” “family's economic status,” “the experience of the toothache during the past year,” “the oral health status,” “the frequency of tooth brushing,” “flossing,” “stress,” and “BMI” were influential factors in tooth decay (*P* <0.05).

**Conclusion:**

Students who had improper BMI, more stress, less educated fathers, families with poor economic status, the experience of toothache within the past year, poor oral health status, and those who used toothbrushes and floss to a minimal degree suffered more tooth decay. Therefore, it is necessary that we pay more attention to these students in designing and implementing educational programs to prevent tooth decay.

## Introduction

One of the most important branches of public health is definitely oral health (1), that according to the definition of the World Health Organization, enables people to speak, socialize and eat without any illnesses or worries (2). It is one of the most important aspects of personal health, which makes it necessary to appraise its significance in the society (3).

Various indicators are used to assess oral health. The DMFT index (The Decayed, Missing, and Filled Teeth) is one of the best epidemiological indicators in dentistry to determine the prevalence and severity of decay which can indicate the oral health status of people (4). In fact, DMFT is a simple, fast, and reliable index in determining oral health (5). In a study conducted in Iran, the prevalence of the decay in deciduous and permanent teeth and also the whole teeth among 7 to 12 year-old-students were 75.2, 41.1, and 89.8%, respectively, and their mean DMFT + dmft was 4.44 (6).

Tooth decay is a multiphase disease whose main causes are: age, socioeconomic factors, poor brushing habits, consumption of harmful beverages, and inappropriate BMI (7–9). The onset and progression of the disease are strongly influenced by the consumption of carbohydrates in the daily diet. Epidemiological studies have also shown that behavioral, social, economic, and clinical factors are associated with the prevalence of tooth decay in children (10). The BMI has been suggested as one of the related factors in tooth erosion (11).

Child obesity seems to have many negative effects on children's oral health (12). Inappropriate eating habits have been suggested as a potential risk factor for tooth decay and obesity. Evidence shows that sugar is involved in tooth decay (13–15) through lowering the pH; it can increase the growth of caries-related microorganisms (16). It has been shown that people with higher sugar intake compared to those who consume less sugar have a significantly higher susceptibility to tooth decay (17–20). Some studies, have shown a direct link between obesity and tooth decay, so that higher BMIs could increase the risk of tooth decay in German children (21). However, showed a contrary relationship between these two indices (22) and some other showed lack of correlation (23, 24). Of course, a systematic review-meta-analysis by Hayden et al. showed that, in general, there is a significant relationship between childhood obesity and tooth decay (25).

Stress has been suggested as one of the contributing factors which will affect students' weight and the general oral health (26). Stress can increase the susceptibility to dental caries by four possible mechanisms. It will affect the immune system by [1] compromising host resistance to cariogenic bacteria (27) reducing salivary secretion which will to decreased clearance (28, 29), [2] unhealthy emotional eating habits followed by frequent snacking and more intake of sugar contained diet (30, 31) and finally [3] impaired implementation of self-care habits (32). Noradrenaline and corticotropin-releasing hormone will also reduce appetite in times of stress; while cortisol is known as an appetite stimulant during stress relief (33). In fact, extensive and complex internal and external factors will affect appetite and consequently, the amount and the type of food consumed by humans. Stress is thought to affect human eating habits (34). In this regard, the study of Mejía-Rubalcava showed that students with moderate or high stress were at higher risk for tooth decay than students with low stress (34). Nevertheless, in the study of Panagiotou et al., no relationship was found between tooth decay and stress in children (35).

Understanding and controlling risk factors are very important in preventing tooth decay and stopping or slowing their progression (36). The prevalence of overweightness and obesity in childhood is also increasing (37). The experience of the stress plays an important role in increasing future physical, psychological and social problems in children. Thus, it is vital that families, teachers, and professional groups such as nurses recognize the stress in children (38). Also, studies on the relationship between tooth decay with stress and obesity have inconsistent and different results. Therefore, this study aimed to determine the relationship between tooth decay with stress and BMI in children in Qazvin.

## Methods

This was a cross-sectional study conducted among 350 students who were referred to the clinic of the faculty of dentistry at Qazvin University of Medical Sciences during 2021–2022.

In this study, samples were selected through convenience sampling method. For this reason, 350 children referred to the clinic of the faculty of dentistry at Qazvin University of Medical Sciences, were selected and entered into the study after taking into account the inclusion criteria, obtaining informed consent, and providing full explanations about the study process. According to the results of the pilot study among 30 students (considering *r* = 0.15 for the correlation between BMI and tooth decay) and also using the table of the sample size for correlation researches, the minimum sample size required was estimated to be 175 people (39). Then, with design effect = 1.9 (DE), the sample size was calculated to be 332 people. Finally, considering the probability of 10% drop in the samples, 365 people were included in the study.

The inclusion criteria were studying in the primary school in Qazvin, referring to the clinic of the faculty of dentistry at Qazvin University of Medical Sciences with the age range of 6–12 years old, understanding Persian language, and willing to participate in the study. Also, lack of cooperation during the study, having a mental disorder, and incompletely answering to the questionnaire were considered as exclusion criteria.

A two-part questionnaire was used to collect data:

A) Demographic questionnaire including questions about age, educational level, educational status, mother's education level, mother's job, father's education level, father's job, family economic status, breastfeeding in infancy, regular weekly exercise, regular walking, experience of toothache in the past year, oral health status, frequent use of toothbrushes per day, and flossing per day.

In addition, the weight of students was measured and recorded using Seca brand scales, without shoes, with the least clothing, and with an accuracy of 0.1 kg. Their height was then measured and recorded using a non-elastic tape measure mounted on the wall, with an accuracy of 0.5 cm, without shoes, in a position that students stood upright and looked straight. Then BMI was calculated by dividing students' weight in kg by their height squared in meters. It should be noted that according to the recommendation of the WHO, BMI <18.5 was considered as low weight, between 18.5 to 24.9 as normal, between 25 to 29.9 as overweight, and equal and above 30 as obese (40, 41). In order to calculate DMFT, filled, decayed, and missing teeth were counted and recorded.

B) To assess children's stress, the 24-item questionnaire, the CSSS made by Sharrer and Ryan-Wenger (42) was used. This questionnaire examines stress-related experiences among children aged 7–12 years old with eleven symptoms related to emotional-cognitive symptoms and thirteen symptoms related to physical symptoms. Accordingly, the score “One” is considered as the existence of the symptom and the score “zero” as the absence of it. The two specifications of “nausea and vomiting” and “grieving” were added after having reviewed other studies, which in total makes 26 items under question. Stress scores ranged from zero to 26 and the presence of stress was reported at three levels; namely, low, medium and high (43). The reliability of the CSSS was evaluated and confirmed in a study by Skybo and Buck with a Cronbach's alpha coefficient of 0.88 (44). The questionnaire used by Valizadeh et al. was also translated into Persian and the validity of both the translation and the content were examined and with the Cronbach's alpha coefficient of 0.76 (45). In the present study, the Cronbach's alpha coefficient was calculated to be 0.84.

Regarding ethical considerations in this research, first the research project number was received from the Vice Chancellor for Research and Technology at Qazvin University of Medical Sciences (with ethics code IR.QUMS.REC.1396.486). Having collected the data, they were entered into SPSS 23 and analyzed by descriptive statistics and logistic regression. It should be noted that entering variables was simultaneously performed through the method of Generalized Linear Models (GLM) with Binary logistic regression response and the last class of variables was selected as the reference class.

## Results

Of all these, 42% (147) of students reported their father's education at the level of diploma, while 76% (266) were within the first to third grade of education. Among all these, only 6.6% (23 people) reported excellent oral health status. Table 1 shows the other demographic (qualitative) characteristics of the students studied. The results also showed that the prevalence of tooth decay among participants was 76.9% (269 people).

**Table 1:** Frequency distribution of students in terms of demographic variables.

**Variable**	**Category**	**Frequency**	**Percentage**
Father's level	Under diploma	136	38.9
of education	Diploma	147	42
	Associate Degree	38	10.9
	Bachelor's degree and higher	29	8.2
Grade	First to third	266	76
	Fourth to sixth	84	24
Oral health status	Excellent	23	6.6
	Good	112	32
	Medium	203	58
	Weak	12	3.4
Flossing	Yes	283	80.9
	No	67	19.1
Frequency of	Twice a day or more	45	12.9
tooth brushing	Less than twice a day	207	59.1
	Sometimes	98	28
The child's	Excellent	238	68
educational status	Good	87	24.9
	Medium	25	7.1
Mother's job	Housekeeper	304	86.9
	Employed	46	13.1
Mother's level	Under diploma	112	32
of education	Diploma	164	46.9
	Associate Degree	28	8
	Bachelor's degree and higher	46	13.1
Family economic	Excellent	26	7.4
status	Good	115	32.9
	Medium	186	53.1
	Weak	23	6.6
Breastfeeding in	Yes	320	91.4
infancy	No	30	8.6
Regular weekly exercise	Yes	180	51.4
	No	170	48.6
Regular walking	Yes	152	43.4
	No	198	56.6
Experience of toothache	Yes	274	78.3
in the past year	No	76	21.7
Mother's job	Employed	274	78.3
	Unemployed	39	11.1
	Retired	37	10.6
Gender	Girl	187	53.4
	Boy	163	46.6

Table 2 shows the mean and standard deviation of other variables among the students being studied. The results showed that the mean and the standard deviation of stress scores among participants were 6.85 ± 4.01 which was at a low level. Also, the mean and standard deviation of the BMI among all participants were 22.78 ± 5.28, so that 17.7% (62 people) were lean, 48.3% (169 people) normal, 24.3% (85 people) were overweight, and 9.7% (34 people) were obese.

**Table 2:** Mean and standard deviation of other variables among the studied students.

**Variable**	**Minimum**	**Maximum**	**Mean**	**SD**
Child age	6.00	12.00	8.50	1.23
Stress	0.00	22.00	6.85	4.01
BMI	9.01	27.05	16.59	3.12

Table 3 shows the results of logistic regression to determine the factors affecting tooth decay among students. As the results show, the variables of father's level of education, family economic status, experience of toothache in the past year, oral health status, frequency of tooth brushing, flossing, stress, and BMI were effective factors in tooth decay (*P* <0.05):

- “Father's level of education” was one of the factors affecting tooth decay, so that the probability of tooth decay in children whose fathers had under diploma or diploma degree was 2.409 and 1.129 times compared to those whose fathers had a bachelor's degree or higher, respectively.
- “Family economic status” was one of the factors affecting tooth decay, so that the probability of tooth decay in children whose family economic status was excellent and fine, was 0.378 and 0.469 times compared to those whose family economic status was poor, respectively.
- The variable of “experience of toothache within the past year” was one of the factors affecting tooth decay, so that the probability of tooth decay in children who had experienced toothache within the past year was 1.458 times compared to those without experiencing toothache at the same year, respectively.
- “Oral health status” was one of the factors affecting tooth decay, so that the probability of tooth decay in children with excellent and fine oral health status was 0.283 and 0.318 times compared to those with poor oral health status, respectively.
- “Frequent use of the toothbrush” was one of the factors affecting tooth decay, so that the probability of tooth decay in children whose daily brushing was “twice or more” or “fewer than twice a day” was 0.327 and 0.555 times compared to those who occasionally used toothbrushes, respectively.
- “Flossing” was one of the factors affecting tooth decay, so that the probability of tooth decay in children who used flossing was 0.551 times compared to those who did not floss.
- The variables of “Stress” and “BMI” were other factors affecting tooth decay, so that through increasing the score of these variables by one unit, the probability of students' tooth decay increased by 1.211 and 1.104 times, respectively. In addition, other demographic variables had no effect on tooth decay (*P* > 0.05).

**Table 3:** Factors affecting tooth decay among students in the test of GLM with Binary logistic regression response.

**Parameter estimates**			
**Variable**	**Category**	**OR (95% CI)**	**Sig**.
Mother's level of education	Under diploma	0.392 (0.057–2.621)	0.632
	Diploma	0.932 (0.513–1.699)	0.146
	Associate degree	1.113 (0.135–9.018)	0.131
	Bachelor's degree and higher		
Mother's job	Housekeeper	1.097 (0.011–0.179)	0.161
	Employed		
Father's level of education	Under diploma	2.409 (0.712–6.017)	0.011
	Diploma	1.129 (1.031–1.906)	0.003
	Associate degree	0.757 (0.671–0.950)	0.348
	Bachelor's degree and higher		
Father's job	Employed	0.618 (0.310–1.224)	0.983
	Unemployed	0.284 (0.079–1.182)	0.073
	Retired		
Family economic status	Excellent	0.378 (0.078–1.806)	0.015
	Good	0.469 (0.416–1.611)	0.042
	Medium	1.104 (0.699–1.981)	0.466
	Weak		
Child's educational status	Excellent	0.334 (0.211–1.340)	0.101
	Good	0.461 (0.127–1.747)	0.166
	Medium		
Grade	First to third	1.042 (0.848-1.350)	0.266
	Fourth to sixth		
Breastfeeding in infancy	Yes	0.583 (0.207–1.648)	0.965
	No		
Regular weekly exercise	Yes	1.527 (0.744–2.065)	0.319
	No		
Regular walking	Yes	0.332 (0.202–1.331)	0.474
	No		
Experience of toothache in the past year	Yes	1.457 (0.702–1.276)	0.018
	No		
Oral health status	Excellent	0.283 (0.068–1.171)	0.010
	Good	0.318 (0.105–0.976)	0.019
	Medium	0.617 (0.553–1.722)	0.335
	Weak		
Frequency of tooth brushing	Twice a day or more	0.327 (0.084–1.266)	0.036
	Less than twice a day	0.555 (0.455–1.135)	0.041
	Sometimes		
Flossing	Yes	0.551 (0.399–1.397)	0.025
	No		
Gender	Girl	0.541 (0.391–1.378)	0.254
	Boy		
Child's age		0.553 (0.408–1.407)	0.132
BMI		1.211 (0.966–1.349)	0.035
Stress		1.104 (0.703–1.981)	0.048
(Intercept)		−207.253	0.373

## Discussion

The results of the present study showed that the prevalence of tooth decay among the participants was 76.9%, which was at a high level. One of the possible reasons for this high prevalence can be the sampling at the clinic level because often people who have dental problems usually go to medical centers. Therefore, the prevalence of tooth decay among these people is higher than the samples available in school the findings of this part are consistent with the results of various studies (36, 45–48). Also, this rate has been reported in some studies (49–51), inconsistent with the findings of the present study. Possible reasons for this discrepancy were as follows; the difference at the age of the children in these studies and the present study, differences in oral health status and dental care between cultures, cities, villages, and countries around the world.

The results of the present study showed that “stress” was at a low level among the participants. One of the possible reasons for the low-level stress in the present study could be the completion of the stress questionnaire by parents, since sometimes children's internal states under stress may be ignored by parents. In line with the present study, a study by Talbot et al. reported the prevalence of stress at a low level (52). The results of the studies of Tanganelli et al. (53) and Calais et al. (54) were not in line with the results of this part of the present study. Possible reasons for this discrepancy may be due to the differences in factors such as the age of children and the stress assessment tools in these studies compared to the present study.

The results of the present study indicated that the mean BMI among the participants was within the normal range. In this regard, it can be said that the majority of them probably had healthy eating habits and tried to do physical activities such as walking or exercise even during a coronavirus pandemic. The approach of parents and their educators in the field of proper nutrition can also be effective in this regard. These results were consistent with the results of different studies (41, 55–58), In terms of BMI in all the above studies, the majority of people were within the normal range.

The results of the present study revealed that the “father's level of education” was one of the factors affecting tooth decay. This part of the results was consistent with the results of various studies (41, 48, 49, 59–62). The results of the present study also indicated that the family economic status was one of the factors affecting tooth decay. The results of various studies (63–66) are consistent with this results. In justifying the possible reasons for these two results, it can be pointed out that as the “father's level of education” increases, his level of awareness enhances. Also, with the increase in the “father's level of education,” the family's economic status will probably improve and the rate of the visits to the dentist for examination will consequently increase. Finally, the combination of these three factors; namely, higher awareness, better economic status and more visits to the dentist, will improve the oral health status of the children.

The results of the present study showed that the experience of “toothache during the past year” was one of the factors affecting tooth decay. This result may indicate that factors such as adopting oral health behaviors and properly educating them to children, taking the initial pain reported by children seriously and acting timely by parents can reduce the rate of primary decay. This part of the results was consistent with the results of various studies (66–69).

The results of the present study showed that the state of oral and dental hygiene was one of the factors affecting tooth decay. This part of the results was consistent with the results of various studies (70–72). Also, the frequency of using a toothbrush and using dental floss were other factors affecting tooth decay. This part of the findings is consistent with the results of various studies (66–69, 73–75). In this regard, it can be said that the above-mentioned three findings are expected to a large extent and indicate the vital and important role of adopting oral and dental hygiene behaviors in creating healthy teeth.

As the results of the present study revealed, stress was one of the factors affecting tooth decay. The results of various studies (33, 76–80) indicate this finding and Panagiotou et al. (35), no relationship was observed between stress and tooth decay in children. In justifying this discrepancy, we can point to possible differences in the rate of tooth decay and stress, as well as differences in stress measuring tools and indices used in these two studies compared to the present study.

The results of the present study indicated that the BMI was one of the factors affecting tooth decay, so that students with higher BMI had more decayed teeth, consequently, it can be said that these children probably had unhealthy eating habits than others, so the risk of tooth decay was higher among them. Similar to these results, this part of the results was consistent with the results of various studies (80–83).

In addition, the results of some studies (56–58) were in contradiction with the results of this part. Among the possible reasons for this contradiction, we can point out the decrease in the intake of necessary minerals such as calcium, which itself can be rooted in causes such as poor economic status and children's nutritional problems.

It seems that the present study is the first one that has simultaneously measured the effect of two variables of the body mass index and stress on tooth decay. It is suggested that the results of this study be used in designing interventions to prevent tooth decay among students.

## Conclusion

Students who had higher BMI, stress, less educated fathers, families with poor economic status, experience of toothache in the past year, poor oral health status, and those who used less toothbrush and floss, showed more tooth decay. Therefore, it is necessary to pay more attention to these students in designing and implementing educational programs to prevent tooth decay.

### Limitations

The target group in this study was students referred to the pediatric dental clinic of the faculty of dentistry at Qazvin University of Medical Sciences. Hence, the results of this study cannot be generalized to other groups of students. Consequently, it is recommended that further studies be conducted among students in other cities as well as different groups of students (in terms of education, gender, age, and place of residence). One of the important limitations of this study was due to the Covid-19 pandemic and school closures, which allowed sampling through convenience sampling method in the pediatric dentistry clinic. Furthermore, the relatively small number of samples and self-reported data collection were among other limitations of this study.

## Data availability statement

The datasets presented in this article are not readily available because they were completed anonymously. Requests to access these datasets should be directed to peimanpanahi63@yahoo.com.

## Ethics statement

The studies involving human participants were reviewed and approved by Ethics Committee of Qazvin University of Medical Sciences. Written informed consent to participate in this study was provided by the participants' legal guardian/next of kin.

## Author contributions

The substantial contributions of the present study to the conceptual design of the work: FH, LD, and RP. The acquisition, analysis, and interpretation of data: RP. The creation of new software used in the work: FH, EK, BO, and LD. Have drafted the work or substantively revised it: FH, RP, and EK. All authors have read and approved the manuscript.

## Conflict of interest

The authors declare that the research was conducted in the absence of any commercial or financial relationships that could be construed as a potential conflict of interest.

## Publisher's note

All claims expressed in this article are solely those of the authors and do not necessarily represent those of their affiliated organizations, or those of the publisher, the editors and the reviewers. Any product that may be evaluated in this article, or claim that may be made by its manufacturer, is not guaranteed or endorsed by the publisher.
